# Quantitative kinetics of intracellular singlet oxygen generation using a fluorescence probe

**DOI:** 10.1038/s41598-020-67155-7

**Published:** 2020-06-30

**Authors:** Kazutoshi Murotomi, Aya Umeno, Sakiko Sugino, Yasukazu Yoshida

**Affiliations:** 1 Molecular Neurophysiology Research Group, Biomedical Research Institute, National Institute of Advanced Industrial Science and Technology (AIST), 1-1-1 Higashi, Tsukuba, Ibaraki, 305-8566 Japan; 2 Health Research Institute, National Institute of Advanced Industrial Science and Technology (AIST), 2217-14 Hayashi-cho, Takamatsu, Kagawa, 761-0301 Japan; 3 LG Japan Lab Inc., Glass Cube Shinagawa 2F, 4-13-14, Higashi Shinagawa, Shinagawa-ku, Tokyo 140-0002 Japan

**Keywords:** Cellular imaging, Fluorescence imaging, Time-lapse imaging

## Abstract

Singlet oxygen (^1^O_2_) is a type of reactive oxygen species involved in numerous physiological activities. We previously reported that ^1^O_2_-specific oxidation products are increased in patients with prediabetes, suggesting that measurement of ^1^O_2_ may be an important indicator of physiological and pathological conditions. The turnover in the generation and quenching of ^1^O_2_ is extremely rapid during biological activities owing to it high reactivity and short lifetime in solution. However, the dynamic changes in ^1^O_2_ generation in living cells have not been fully explored. In this study, we investigated whether the kinetics of ^1^O_2_ generation can be quantified using a far-red fluorescent probe for mitochondrial ^1^O_2_, Si-DMA, following addition of the ^1^O_2_ generator, endoperoxide, to mammalian cells. The kinetics of Si-DMA fluorescence intensity dose-dependently increased following treatment of mammalian living cells with endoperoxide. Alternatively, treatment with ^1^O_2_ quenchers decreased the fluorescence intensities following endoperoxide treatment. Our results indicate that the kinetics of intracellular ^1^O_2_ can be readily obtained using Si-DMA and time-lapse imaging, which provides new insights into the mechanism of ^1^O_2_ generation in mammalian cells and the exploration of ^1^O_2_ generators and quenchers.

## Introduction

Reactive oxygen species (ROS) play critical roles in host defence and the production of biologically essential substances, as well as in the regulation of physiological functions as redox signalling messenger^1^. Singlet oxygen (^1^O_2_) is a type of ROS that is involved in numerous biological processes^2^ and has also been applied in water disinfection and photodynamic therapy (PDT) in cancer treatment. PDT can effectively kill cancer cells via the reaction between a photosensitiser and laser light, as ^1^O_2_ induces cytotoxicity due to the strong oxidation reaction^3,4^. Our previous studies demonstrated that the levels of ^1^O_2_-specific oxidation products 10- and 12-(*Z,E*)-hydroxyoctadecadienoic acids (HODEs) transiently increased in a mouse model of type 2 diabetes mellitus (T2DM)^5^, and that plasma levels of 10- and 12-(*Z,E*)-HODEs were positively correlated with glucose levels in patients with prediabetes^6,7^. In addition, the production of cytoplasmic ^1^O_2_ induces cell cycle progression in HeLa cells, whereas the nuclear production delays the cell cycle^8^. These findings suggest that ^1^O_2_ may contribute to the regulation of physiological and pathological conditions in mammals.

Singlet oxygen is produced by the energy transfer of triplet oxygen, which constitutively occurs in plant leaves during the reaction between light and chlorophyll pigments in cells^9^. In mammals, ^1^O_2_ is physiologically generated in the skin upon exposure to ultraviolet-A^10^ or as a product of the reaction between hypochlorous acid and hydrogen peroxide mediated by myeloperoxidase^11,12^. As ^1^O_2_ generation in mammalian cells is essential for biological activity, methods for its precise detection and quantification are needed to facilitate research for better understanding its roles in physiological and pathological conditions^13,14^. One of the primary methods for ^1^O_2_ detection is the measurement of near-infrared phosphorescence at 1270 nm^15^; however, this phosphorescence signal is narrow due to weak emission intensities. Further, this platform requires the use of sophisticated instruments^16^. Fluorescence probes with high sensitivity, fast response time, and high spatial resolution under microscopic imaging^17^ have also been developed for the detection of ^1^O_2_, several of which even enable ^1^O_2_ imaging in living cells^18–22^. Recently, a far-red fluorescent probe for ^1^O_2_ composed of 9,10-dimethylanthracene (DMA) and silicon-containing rhodamine (Si-rhodamine) moieties, namely Si-DMA, was developed for monitoring ^1^O_2_ at subcellular levels^23^. One of the main advantages of Si-DMA over other real-time probes is its increased sensitivity to specifically detect mitochondrial ^1^O_2_ at the subcellular level^19–22^. This probe can react with mitochondrial-originating ^1^O_2_^23^ as the Si-rhodamine contained in Si-DMA accumulates in the mitochondria, and the diffusion distance of intracellular ^1^O_2_ reaches a maximum of approximately 300 nm in aqueous solution^24^. Although the turnover in the generation and quenching of ^1^O_2_ is predicted to be very rapid for the maintenance of cellular functions, the detailed dynamic changes in intracellular ^1^O_2_ generation levels in living cells remain largely unknown.

In this study, we investigated the feasibility of using the ^1^O_2_ fluorescence probe Si-DMA to obtain kinetic data on intracellular ^1^O_2_ generation and quenching following treatment of cells with an ^1^O_2_ generator, endoperoxide. Toward this end, we treated the mouse fibroblast cell line, NIH3T3, and the human hepatocarcinoma cell line, HepG2, with the ^1^O_2_ generator endoperoxide, or the oxygen quenchers, sodium azide (NaN_3_) and astaxanthin, at various concentrations, and measured the resulting fluorescence intensities of Si-DMA with fluorescence time-lapse imaging. HepG2 cells were used as a common *in vitro* liver model since ^1^O_2_ was reported to be produced in the liver^25,26^. Astaxanthin is a carotenoid pigment with high ^1^O_2_-quenching capacity *in vitro*^27–30^, and was therefore used as a model for evaluating the feasibility of detecting ^1^O_2_ quenching of food ingredients in living cells. This technique may offer a valuable tool to better understand the mechanism of ^1^O_2_ generation, while providing a practical method to rapidly and simply screen for candidate ^1^O_2_ generators or quenchers in mammalian cells.

## Results

### Dynamic changes in Si-DMA fluorescence after endoperoxide treatment

**Figure 1:**
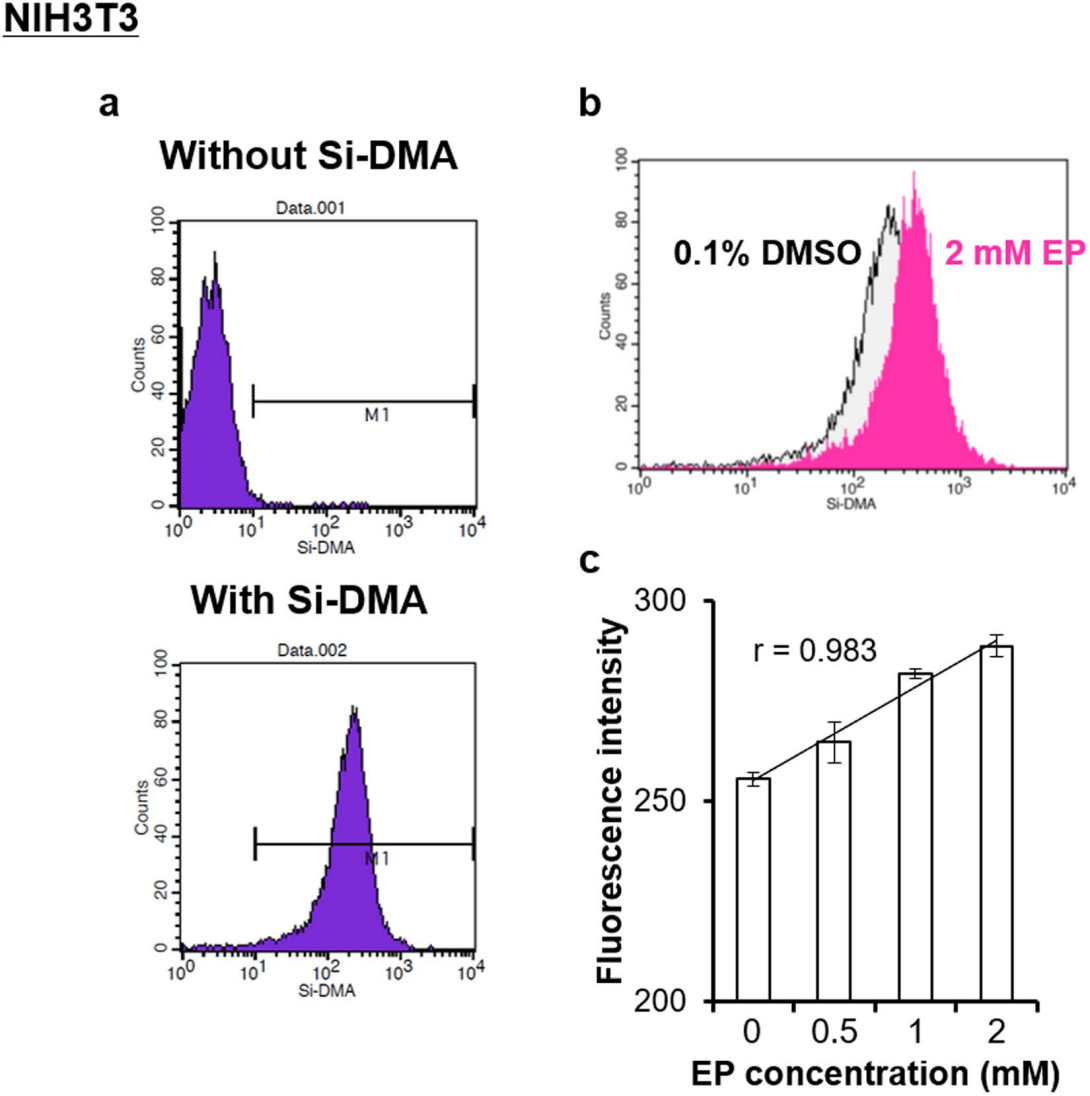
Measurement of Si-DMA fluorescence using FACS analysis. (**a**) The difference in fluorescence intensities in NIH3T3 mouse fibroblasts with and without Si-DMA. The intensity is indicated as the mean value of the M1 range. (**b**) Relationship between the number of cells and Si-DMA fluorescence intensity after 1% DMSO or 2 mM endoperoxide (EP) treatment. (**c**) Averages of Si-DMA fluorescence intensity after EP treatment. Results are expressed as mean ± standard deviation (n = 2).

We first attempted to quantify the intracellular ^1^O_2_ concentration based on Si-DMA fluorescence intensities, which react exclusively with mitochondrial-originating ^1^O_2_^23^ as the diffusion distance of intracellular ^1^O_2_ is less than 300 nm in aqueous solution^24^, using fluorescence-activated cell sorting (FACS) (Fig. 1a). We used an ^1^O_2_ generator endoperoxide, which is commercially available from WakenBtech Co., Ltd. (Osaka Japan), contains an epidioxy group crosslinked in the aromatic hydrocarbon, and generates ^1^O_2_ during thermolysis without photosensitiser (Equation 1). As this material produces ^1^O_2_ via thermal decomposition at temperatures greater than 25 °C^27^, we maintained the storage temperature of endoperoxide under 20 °C until just before use to avoid decomposition.1

The intensities were measured after the addition of endoperoxide at concentrations ≤2 mM because the use of higher concentrations resulted in insoluble endoperoxide in the culture medium at 37 °C. In FACS analysis, the fluorescence intensity in NIH3T3 cells clearly increased after treatment of 1 or 2 mM endoperoxide compared with that of the control cells treated with 1% dimethyl sulfoxide (DMSO) (Fig. 1b,c). Increase in fluorescence intensity was observed for endoperoxide, with a dose-dependent increase up to 2.0 mM, at which point it reached a plateau (correlation coefficient = 0.983, Fig. 1c). Although the addition of hydrogen peroxide and hypochlorous acid to cells was attempted as a method for generation of ^1^O_2_ in the culture medium, reproducible results were not obtained (data not shown).

**Figure 2:**
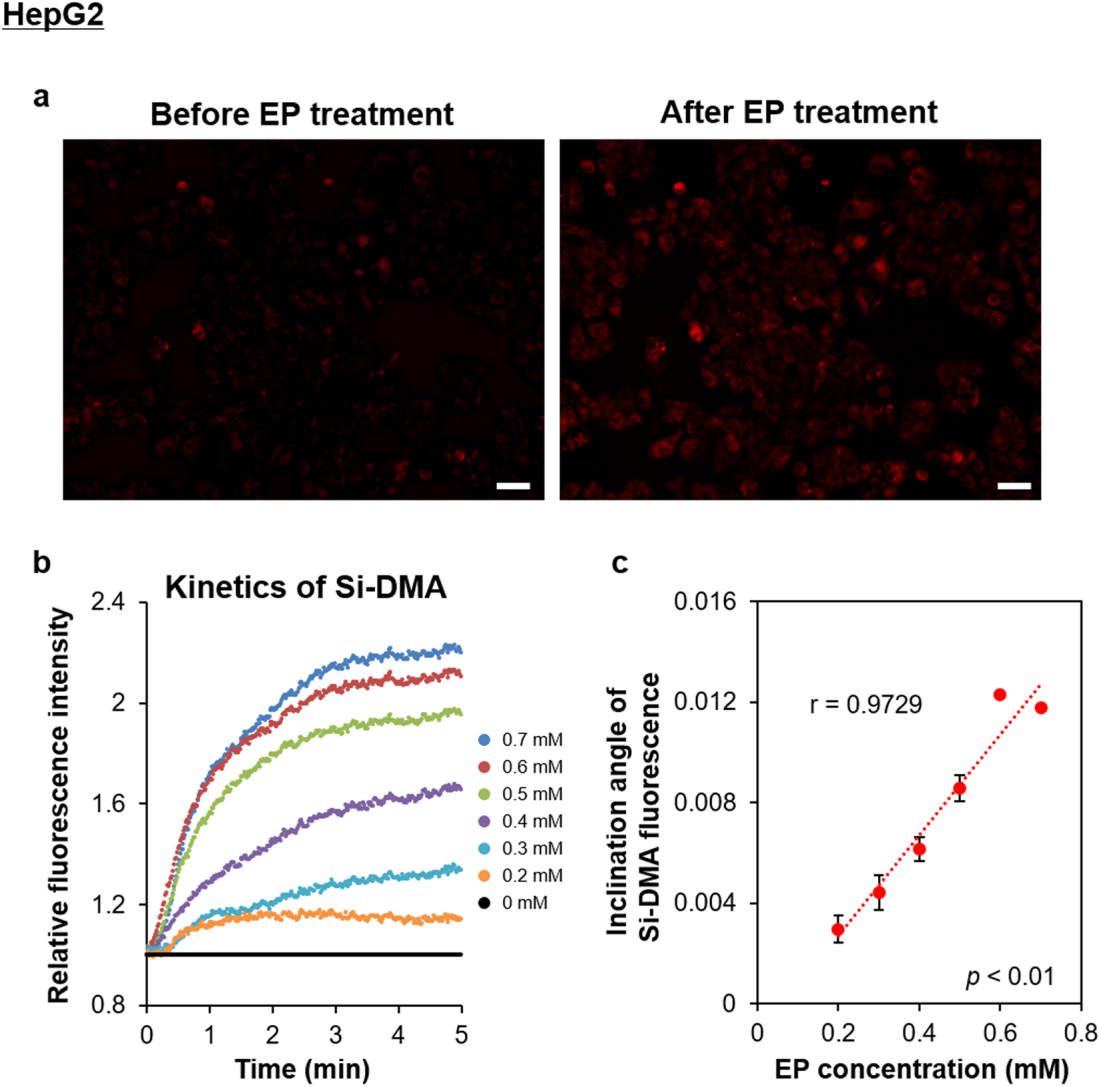
Dynamic changes in Si-DMA fluorescence intensity using time-lapse imaging. (**a**) Representative fluorescence microscopic images in HepG2 cells incorporating Si-DMA before and after endoperoxide (EP) treatment, respectively. Scale bars = 50 μm. (**b**) Kinetics of relative Si-DMA fluorescence intensities after EP treatment. The intensities were obtained from each sequence of images (movie) and relative intensities were normalised to the control values (1% DMSO treatment). (**c**) Correlations between the inclination angle of Si-DMA fluorescence and EP concentration. The inclination angle represents the degree of the slope for the linear regression of Si-DMA fluorescence intensity against the EP concentration. Results are indicated as mean ± SE (n = 3). The strength of the association between two parameters was evaluated on the basis of Pearson’s correlation coefficient.

In addition, we observed alterations of fluorescence intensity in HepG2 cells by fluorescence microscopy before and after endoperoxide treatment (Fig. 2a). Time-lapse imaging showed that the Si-DMA fluorescence intensities markedly increased in an endoperoxide concentration-dependent manner from 0.2 to 0.6 mM, reaching a plateau at 0.7 mM endoperoxide within 10 min after treatment (Fig. 2b). The slope of relative fluorescence intensity after endoperoxide treatment showed a strong relationship between the inclination angle and endoperoxide concentration (p < 0.01, r = 0.9729, Fig. 2c).

These results indicate that the relative ^1^O_2_ yield in living cells could be quantitatively measured using Si-DMA after treatment of 0.5–2 mM endoperoxide with FACS or 0.2–0.7 mM endoperoxide with fluorescence microscopy. Singlet oxygen becomes rapidly quenched in culture medium and mammalian cells as the lifetime of ^1^O_2_ is less than 3 μs in cells cultured in a H_2_O-based medium^2^, and 15 μs in cells cultured in a D_2_O-based medium^31^. In addition, ^1^O_2_ generated by endoperoxide treatment readily reacts with mitochondrial molecules, such as membrane lipids^32,33^ and glutathione^34^. Thus, we considered that time-lapse imaging using fluorescence microscopy is an appropriate method for measurement of the dynamic changes in ^1^O_2_ yield in living cells.

### Measurement of Si-DMA fluorescence intensity after treatment of ^1^O_2_ quenchers

**Figure 3:**
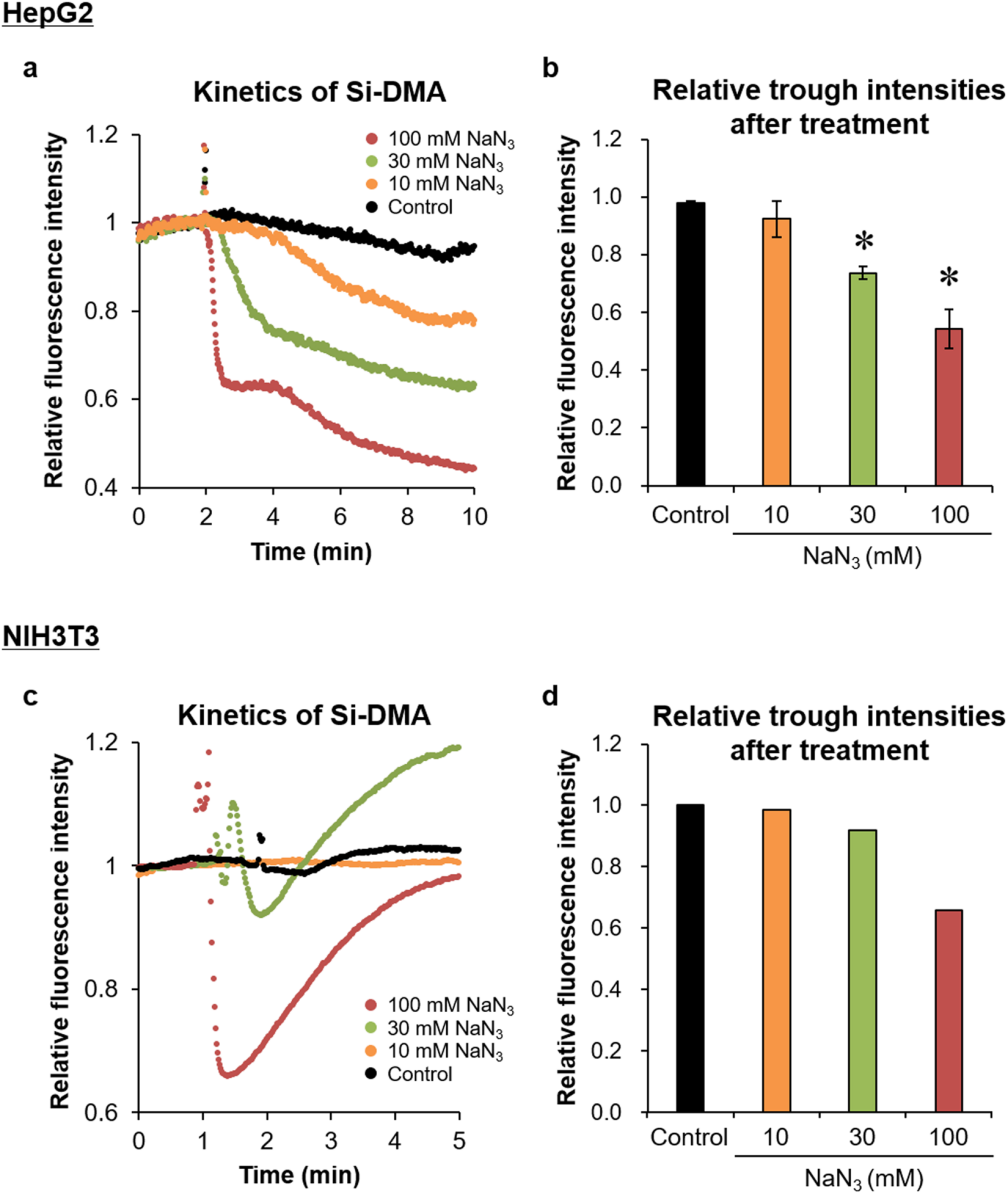
Effect of the ^1^O_2_ quencher NaN_3_ on Si-DMA fluorescence intensity. (**a,c**) Kinetics of the intensity in HepG2 (**a**) and NIH3T3 (**c**) cells after NaN_3_ treatment. Relative fluorescence intensity is presented as the average values relative to those obtained during 1 min before NaN_3_ treatment, set to 1. (**b,d**) Comparison of trough intensity in Si-DMA fluorescence within the first 3 min after NaN_3_ treatment. Relative intensities were determined by the ratio of trough intensity to the average obtained during 1 min before NaN_3_ treatment. Results are indicated as mean ± SE (b: n = 3, d: n = 1). *p < 0.05 compared with the control.

To evaluate whether decreases in ^1^O_2_ generation can also be observed based on Si-DMA fluorescence, we treated HepG2 cells with the ^1^O_2_ quencher NaN_3_ after confirmation of the increase in fluorescence intensity with 0.5 mM endoperoxide treatment. As shown in Fig. 3a, the fluorescence intensity dose-dependently decreased immediately after NaN_3_ treatment. These decreases did not appear to be due to direct cellular damage, as the viability of HepG2 cells was not reduced at 24 h after 10 mM or 30 mM NaN_3_, whereas treatment with more than 60 mM NaN_3_ decreased the viability of HepG2 cells (Supplementary Fig. S1). In addition, the relative trough intensity after NaN_3_ treatment significantly decreased compared with that of the control group (Fig. 3b). Interestingly, the kinetics of fluorescence intensity after NaN_3_ treatment varied according to experimental conditions with a transient decrease in Si-DMA fluorescence intensity observed in NIH3T3 cells even after 0.1 mM endoperoxide and 100 mM NaN_3_ treatment (Fig. 3c,d). With treatment of 30 mM NaN_3_, the intensity tended to exceed the initial value after the transient reduction (Fig. 3c). These results indicate that quenching of ^1^O_2_ in living cells can be observed by time-lapse imaging with Si-DMA and fluorescence microscopy.

**Figure 4:**
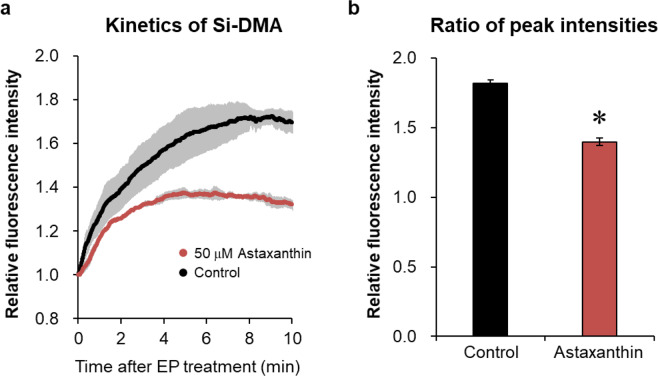
Effect of astaxanthin on Si-DMA fluorescence intensity. (**a**) Kinetics of the fluorescent intensity in HepG2 cells after 0.5 mM endoperoxide (EP) treatment. At 24 h after 50 μM astaxanthin treatment, Si-DMA was added, and the fluorescence intensity was measured. Relative fluorescence intensity was calculated against the value under EP treatment, set to 1 (grey bar = SE, n = 3). (**b**) Comparison of peak intensity in Si-DMA fluorescence after 0.5 mM EP treatment. Relative intensities were determined by the ratio of peak intensity to the value obtained just after EP treatment. Results are indicated as mean ± SE (n = 3). *p < 0.05 compared with the control.

As shown in Fig. 4a, the Si-DMA fluorescence in HepG2 cells clearly decreased after treatment with 50 μM astaxanthin (which was confirmed to have no cytotoxic effect in preliminary experiments; data not shown) following 0.5 mM endoperoxide treatment compared with that in the control cells treated with 1% DMSO. The peak values of the fluorescence intensity in the control and astaxanthin groups were 1.82 and 1.40, respectively (Fig. 4b). These results suggest that this method could be applied to investigate the ^1^O_2_-quenching capacity of various materials in living cells.

## Discussion

Due to its short lifetime and low concentration in mammalian cells, it is difficult to quantify intracellular ^1^O_2_. Although some studies recently demonstrated that ^1^O_2_ in living cells could be detected using fluorescent probes^18–22^, the dynamic alterations in intracellular ^1^O_2_ generation have remained unclear. In the present study, we developed and verified a method for the quantitative measurement of ^1^O_2_ generation in living cells using the fluorescent probe Si-DMA.

Endoperoxide solution stably generated measurable amounts of ^1^O_2_ in the culture medium, whereas hydrogen peroxide and hypochlorous acid produced non-reproducible amounts of intracellular ^1^O_2_. Using the endoperoxide solution and Si-DMA, we observed an increase in ^1^O_2_ generation that positively correlated with the endoperoxide concentration. In contrast, the intracellular ^1^O_2_ generation decreased after ^1^O_2_ quencher treatment. In particular, the fluorescence intensities were detectable despite the rapid changes in the intracellular ^1^O_2_ generation and quenching, which occurred within a few minutes. These observations can be explained by the high selectively of the probe Si-DMA toward ^1^O_2_. The structure of Si-DMA allows photoinduced electron transfer (PET) between dimethylanthracene (DMA, electron donor) and Si-rhodamine (electron acceptor) by light excitation. Si-rhodamine is a fluorescence pigment, while DMA acts as a quencher. Hence, the fluorescence of excited Si-rhodamine becomes quenched by intramolecular PET from DMA, resulting in dim fluorescence (ϕ_fl_ = 0.01). The anthracene analogue specifically traps and reacts with ^1^O_2_, which introduces an endoperoxide group to the centre ring of the DMA unit in Si-DMA leading to the formation of Si-DMEP (Equation 2). As a result, PET from DMA to Si-rhodamine is inhibited due to the reduced electron-donating capacity of DMA, and the formed Si-DMEP exhibits an approximately 18-fold higher fluorescence than Si-DMA in methanol^23^. The DMA moiety in Si-DMA does not release ^1^O_2_ following endoperoxidation, which is in accordance with the endoperoxide form of Singlet Oxygen Sensor Green®, SOSG-EP^35^. In contrast, certain anthracene derivatives undergo a reversible reaction with ^1^O_2_; that is, endoperoxide formation is followed by the release of ^1^O_2_^36^. As Si-DMA can selectively and stably react with ^1^O_2_, we could monitor intracellular ^1^O_2_ levels using time-lapse imaging.2

Considering that the lifetime of ^1^O_2_ is approximately 3 μs in the nuclei of H_2_O-incubated cells^24^, the lifetime of ^1^O_2_ in this study was probably shorter since the culture medium contained high levels of amino acids that also react with ^1^O_2_. Moreover, since the diffusion distance of ^1^O_2_ is less than 300 nm in 6 μs^24^ and endoperoxides can diffuse to mitochondria, we speculated that only mitochondrial ^1^O_2_ was detected by Si-DMA in this study. This is also supported by the fact that Si-DMA selectively localizes in the mitochondria at a concentration of 100 nM^23^, which was used in this study. Hence, the results of this study deepen our understanding of the mechanisms underlying the generation of mitochondrial ^1^O_2_. Furthermore, the half-life of endoperoxide is approximately 20 minutes in ethanol/chloroform/D_2_O (50:50:1, v/v/v) at 35 °C^27^; similarly, Si-DMA is considered to be photostable as it has been detected in HeLa cells in at least 15 minutes following treatment^23^. These previous findings suggest that endoperoxide and Si-DMA are likely stable in cells and culture media. Therefore, although ^1^O_2_ was rapidly quenched under the present experimental conditions, we were able to detect mitochondrial ^1^O_2_ using time-lapse fluorescence imaging.

Specific food ingredients, such as astaxanthin and lycopene, are known to exhibit high ^1^O_2_-quenching capacities under cell-free conditions^28,37,38^. It is believed that these ingredients can exert similar quenching effects in living cells, contributing to the prevention of human chronic diseases, including cardiovascular diseases and diabetes^39,40^. Although it was previously reported that the intracellular ^1^O_2_ quenching capacity of β-carotene could not be observed using laser-based time-resolved photosensitised methods^41^, we showed that the fluorescence intensities of Si-DMA in astaxanthin-treated cells significantly decreased, indicating that astaxanthin can quench ^1^O_2_ in mammalian cells. On the other hand, we sought to evaluate whether β-carotene quenches intracellular ^1^O_2_ generated by endoperoxide; however, reproducible results were not obtained, which might be because carotenoids preferentially accumulate in cell membranes. β-carotene is localized deep inside the hydrophobic core membranes and oriented parallel to the membrane surface, whereas astaxanthin, with two polar hydroxyl groups, is anchored across the cell membrane with the polar groups oriented outside the membrane^42^. While the β-carotene reaction sites for ^1^O_2_ are buried in the cell membrane, those of astaxanthin cross the membrane. We, therefore, speculate that astaxanthin localized in the mitochondrial membrane can quench mitochondrial ^1^O_2_ produced by endoperoxide. This further confirms that the ^1^O_2_-quenching capacity of various materials can be evaluated using Si-DMA in time-lapse imaging, offering a novel approach for exploring the modes of action of ^1^O_2_ quenchers in living cells. Furthermore, the present approach might clarify whether some ingredients prevent chronic diseases via ^1^O_2_-quenching.

As the biological function of ^1^O_2_ in mammals remains poorly understood, we expect that the application of the fluorescence probe Si-DMA will help to elucidate the detailed mechanism underlying intracellular ^1^O_2_ generation. Although the plasma levels of the ^1^O_2_-mediated oxidation products 10- and 12-(*Z*,*E*)-HODEs correlated with fasting glucose levels in patients with prediabetes^6,7^ and temporarily increased before the pathogenesis of T2DM in mice^5^, the precise mechanisms behind this increase in a prediabetic state remain elusive. Singlet oxygen itself or its oxidation products, 10- and 12-(*Z*,*E*)-HODEs, may induce an adaptative response to ROS exposure^43^, leading to enhanced cellular detoxification activities, against T2DM. Thus, it is important to elucidate the biological function of ^1^O_2_ and the underlying generation mechanism. We also observed Si-DMA-positive cells in primary murine hepatocytes (data not shown), suggesting that Si-DMA is also applicable for the detection of ^1^O_2_ in animals. Thus, the application of Si-DMA in patients and model mice with T2DM might contribute to a better understanding the role of ^1^O_2_ in the development of T2DM.

In conclusion, we developed a method to quantitatively measure of intracellular ^1^O_2_ levels using a fluorescence probe. To the best of our knowledge, this is the first study assessing the dynamic changes in ^1^O_2_ generation in living cells. This technique may contribute toward understanding the mechanism of ^1^O_2_ generation and can offer a valuable tool for exploring the role of ^1^O_2_ quenchers in living mammalian cells.

## Methods

### Cell culture

Human hepatic carcinoma HepG2 cells and mouse NIH3T3 fibroblasts were grown in Dulbecco’s modified Eagle medium (DMEM; Sigma Aldrich, MO, USA) supplemented with 10% foetal bovine serum (ICN Biochemicals, CA, USA) in a humidified atmosphere containing 5% CO_2_ at 37 °C.

### Measurement of Si-DMA fluorescence intensity

The cells (5 × 10^5^ per well or dish) were seeded in a 6-well plate (Nunc, MA, USA) for flow cytometry or in a 35-mm glass-based dish (Nunc) for live cell imaging. One day after incubation, each concentration (0.5–1.0 mM) of endoperoxide [3-(1, 4-epidioxy-4-methyl-1, 4-dihydro-1-naphthyl) propionic acid; WakenBtech Co., Ltd., Kyoto, Japan] dissolved in DMSO was added to the cells and incubated for 30 min at 37 °C and 5% CO_2_; control cells were treated with DMSO at the same concentrations. The endoperoxide solution was cooled below 20 °C just before use, as endoperoxide stably generates ^1^O_2_ above 35 °C. The cells were washed with serum-free DMEM and incubated in Hanks’ balanced salt solution (HBSS) containing Si-DMA (Dojindo Laboratories, Kumamoto, Japan), which is a suitable fluorescent dye for ^1^O_2_ imaging^23^, for 30 min. Si-DMA was dissolved in DMSO according to the manufacturer’s instruction and stored at –30 °C until used for the experiments. After washing with phosphate-buffered saline, the cells were harvested and analysed by flow cytometry with the FACSCalibur system (Becton Dickinson, NJ, USA). The laser amplitude was appropriately set to divide into two groups (with or without Si-DMA) and the median of the M1 marker was defined as the Si-DMA fluorescence intensity (Fig. 1a).

For live cell imaging, cells seeded onto the 35-mm glass-based dish were washed with HBSS after incubation in Si-DMA-containing solution. Subsequently, 1 ml HBSS was added to the cells and the dish was placed on the stand of the fluorescence microscope (BZ-X710 All-in-one, Keyence, Tokyo, Japan). Fluorescence images were obtained from snapshots of movies (around 1 s exposure) taken for 30 min. At 5 min after filming, endoperoxide or the negative control (1% DMSO) was added to the cell-seeded dish and the fluorescence intensities were continuously measured. The single oxygen quencher NaN_3_ was added to the dish at 5 min after endoperoxide or 1% DMSO treatment. Astaxanthin (Sigma Aldrich) was dissolved in DMSO and added to the cells 1 day before endoperoxide treatment. The fluorescence intensity was analysed using BZ-X Analyzer (Keyence), and the results are presented as the relative fluorescence intensity to that measured just prior to the addition of each material.

### Measurement of cell viability

HepG2 cells were plated in the wells of a 96-well microplate (Nunc) and incubated overnight at 37 °C with 5% CO_2_. DMEM containing each concentration of NaN_3_ was prepared and added to the microplate at 100 μl/well. After 24 h of incubation, Premix WST-1 Cell Proliferation Assay reagent (Takara Bio Inc., Shiga, Japan) was added to each well and the absorbance was measured at 450 nm. Cell viability was calculated relative to the absorbance of control cells set at 100%.

### Statistical analysis

The results are expressed as means ± standard deviation or standard error. Statistical analysis was performed using analysis of variance followed by Tukey’s test for multiple comparisons with Ekuseru-Toukei 2012 software (Social Survey Research Information Co., Ltd., Tokyo, Japan). The strength of correlation between two variables was analysed by Pearson’s correlation coefficient. Differences with a probability of 5% or less were considered statistically significant.

## Supplementary information

Supplementary information.

## Data Availability

The all data sets used and/or analysed during this study are available from the corresponding author on reasonable request.
