# Supplementary material for: Quantitative kinetics of intracellular singlet oxygen generation using a fluorescence probe
Source: Sci Rep. 2020 Jun 30;10:10616. doi: 10.1038/s41598-020-67155-7 (PMC7327044; doi:10.1038/s41598-020-67155-7)
Supplement: Supplementary file 1 — Supplementary information. [file 41598_2020_67155_MOESM1_ESM.docx]

**Supplementary Information**

**Quantitative kinetics of intracellular singlet oxygen generation using a fluorescence probe**

Kazutoshi Murotomi^1*^, Aya Umeno^2^, Sakiko Sugino^2^, Yasukazu Yoshida^2^

^1^Molecular Neurophysiology Research Group, Biomedical Research Institute, National Institute of Advanced Industrial Science and Technology (AIST), Japan

^2^Health Research Institute, National Institute of Advanced Industrial Science and Technology (AIST), Japan

­­***Correspondence:**

Kazutoshi Murotomi, PhD

Biomedical Research Institute, National Institute of Advanced Industrial Science and Technology (AIST), 1-1-1 Higashi, Tsukuba, Ibaraki 305-8566, Japan

TEL: +81-87-869-4207

FAX: +81-29-861-6407

E-mail: [k-murotomi@aist.go.jp](mailto:k-murotomi@aist.go.jp)

**
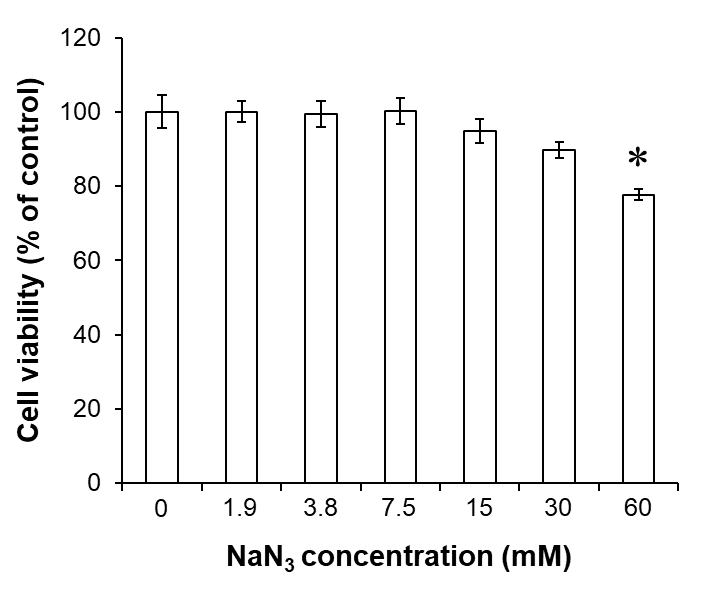
**

Figure S1. Viability of HepG2 cells after treatment with NaN_3_. Results are presented as mean ± SE (n = 3). Cells treated without NaN_3_ were used as the control. **p* < 0.05 compared with control.
